# The chronic kidney disease epidemiology collaboration equation combining creatinine and cystatin C accurately assesses renal function in patients with cirrhosis

**DOI:** 10.1186/s12882-015-0188-0

**Published:** 2015-12-01

**Authors:** Elisabeth Krones, Peter Fickert, Sabine Zitta, Stefan Neunherz, Katharina Artinger, Gilbert Reibnegger, Franziska Durchschein, Doris Wagner, Tatjana Stojakovic, Vanessa Stadlbauer, Günter Fauler, Rudolf Stauber, Gernot Zollner, Daniela Kniepeiss, Alexander R. Rosenkranz

**Affiliations:** Division of Gastroenterology and Hepatology, Department of Internal Medicine, Medical University of Graz, Graz, Austria; Clinical Division of Nephrology, Department of Internal Medicine, Medical University of Graz, Graz, Austria; Department of Cardiology, Klinikum Leverkusen, Leverkusen, Germany; Institute for Physiological Chemistry, Medical University of Graz, Graz, Austria; Division for General Surgery, Department of Surgery, Medical University of Graz, Graz, Austria; Clinical Institute of Medical and Chemical Laboratory Diagnostics, Medical University of Graz, Graz, Austria; Department of Surgery, Division of Transplant Surgery, Medical University of Graz, Graz, Austria

**Keywords:** Cirrhosis, Cystatin C, Glomerular filtration rate, Renal function, Clearance, Sinistrin

## Abstract

**Background:**

Accurate measurement of renal function in cirrhotic patients is still challenging. To find the best test for the determination of the true glomerular filtration rate (GFR) in cirrhotic patients this study prospectively compared measured (m)GFR, the gold standard, with estimated (e)GFR using equations based on serum levels of creatinine and cystatin C.

**Methods:**

GFR was measured by sinistrin clearance using the bolus method in 50 patients with cirrhosis (Child Turcotte Pugh score A, B and C) and 24 age-matched healthy subjects as controls. Measured (m)GFR was compared to eGFR using bias, accuracy 10 % and 30 %, as well as correlation coefficients.

**Results:**

Creatinine-based equations generally overestimated GFR in patients with cirrhosis and showed a bias (average difference between mGFR and eGFR) of −40 (CG), −12 (MDRD) and −9 (CKD-EPI-Cr) ml/min/1.73 m^2^. Cystatin C-based equations underestimated GFR, especially in patients with Child Turcotte Pugh score C (bias 17 ml/min/1.73 m^2^for CKD-EPI-CysC). Of these equations, the CKD-EPI equation that combines creatinine and cystatin C (CKD-EPI-Cr-CysC) showed a bias of 0.12 ml/min/1.73 m^2^ as compared to measured GFR.

**Conclusions:**

The CKD-EPI equation that combines serum creatinine and cystatin C measurements shows the best performance for accurate estimation of GFR in cirrhosis, especially at advanced stages.

**Electronic supplementary material:**

The online version of this article (doi:10.1186/s12882-015-0188-0) contains supplementary material, which is available to authorized users.

## Background

Renal function has a pivotal prognostic value in patients with cirrhosis [1]. Its critical prognostic impact is indicated by the inclusion of serum creatinine (Cr) levels in the widely used MELD (Model for end-stage liver disease) score, whose value is an accurate predictor of 3-month mortality in cirrhosis. As a result, the MELD score is widely used for determining priority for liver transplantation [2]. An accurate evaluation of renal function is of utmost importance in patients with cirrhosis and patients with acute-on-chronic liver failure, especially those being evaluated for transplantation. Although the serum level of Cr is an easily measurable and widely available marker of excretory renal function, it has limitations in assessing glomerular filtration rate (GFR) in patients with cirrhosis [3–6]. Due to malnutrition, low protein intake, decreased Cr synthesis and increased tubular secretion, GFR in patients with cirrhosis is overestimated when estimated by serum Cr levels [3]. The equations that are most frequently used to estimate GFR include Cockcroft Gault (CG) [7], Modification of Diet in Renal Disease (MDRD) [8] and the Chronic Kidney Disease Epidemiology Collaboration formula (CKD-EPI); these equations, however, require corrections for age, gender, ethnicity and body weight. Estimated (e)GFR based on serum levels of cystatin C (CysC) has been claimed to be more accurate than Cr-based equations [9, 10]. CysC is a nonglycosylated low molecular weight protein of the cystatin superfamily of cysteine protease inhibitors [11]. In renal impairment, its levels increase faster than Cr levels, and have been considered as valuable for the early detection of renal dysfunction [12, 13]. Unlike Cr, CysC is independent of muscle mass, age and gender, and not influenced by serum bilirubin or malignancy [3, 4, 11, 14]. However, measurement of CysC, has recently been reported to be influenced by factors such as low serum albumin levels, elevated white blood cell count, and elevated CRP levels [15]. These abnormalities are frequently present in cirrhotic patients and consequently are likely to impair the reliability of CysC-based equations. Therefore, measurement rather than estimation of GFR seems to be mandatory for these patients.

Although technically demanding, time-consuming and costly, inulin clearance has been considered the gold standard for determination of GFR. Inulin is freely filtered by the glomerulus and neither secreted, reabsorbed, synthesized nor metabolized by the kidney [16]. In Europe, sinistrin, an inulin-like polyfructosan, exhibiting the same properties as inulin, is preferentially used for the determination of GFR [17, 18].

The aim of this study was to identify the best equation for the determination of eGFR in cirrhotic patients without concomitant kidney disease or conditions that are prone to structural kidney damage (e.g. diabetes). For that purpose, GFR was determined in 50 cirrhotic patients using sinistrin clearance measured after single injection technique (mGFR, measured GFR) and compared to that estimated by Cr- and CysC-based equations (eGFR). Results obtained from cirrhotics were compared to those of healthy controls.

### Subjects and methods

#### Study population and collection of data

This prospective study was performed in accordance with the Declaration of Helsinki and the applicable laws of the Republic of Austria in 74 patients referred to our center between 2012 and 2014. The study protocol was approved by the ethics committee of the Medical University of Graz (23–497 ex 10/11). No sample size calculation was done since this study was performed as a pilot study. To compare different methods of GFR estimation with GFR measurement, 50 patients with compensated or decompensated cirrhosis of different etiologies as well as 24 age-matched healthy living kidney donors representing the healthy control group were included. Patients with known underlying kidney disease, diabetes, insufficiently treated arterial hypertension, collagen vascular diseases, known malignancy of the urogenital tract, recurrent urinary tract infections, ingesting nephrotoxic drugs such as aminoglycosides, or having a regular intake of non-steroidal anti-inflammatory drugs were excluded; patients who were pregnant, and/or breastfeeding and patients being unable to give informed consent due to cognitive impairment were also excluded. Patients were recruited during their visit to the liver outpatient clinic or while being on the inpatient ward. A written informed consent was obtained from each patient.

##### Measurements

All investigations for the study took place at the Department of Nephrology at the Medical University of Graz. After placing a catheter in the antecubital vein, basement blood samples for serum analyses were taken. Presence of ascites was determined by abdominal ultrasound and patients were clinically checked for hepatic encephalopathy. Determination of mGFR by sinistrin clearance was performed using the bolus method, which has been considered to be advantageous over the continuous infusion since neither urine samples nor steady state conditions are required [17, 19]. We employed a single-injection technique with sufficiently long sinistrin serum concentration contours adapted to a two-compartment kinetic model with variable parameters for transfer- and elimination rates, which was extensively described earlier [18, 20–22]. The computer model used in our study allows a self-validation of the data that in turn results in GFR measurements of very high precision [17, 21–23]. Application of an exogenous marker performed as single injection experiment offers the opportunity of exact clearance determination without potential errors of incorrect steady state conditions or incomplete urinary collection. Each study participant received an injection of 2500 mg of sinistrin (Inutest^®^, Fresenius KABI, Graz, Austria) intravenously. Serum concentrations of sinistrin were determined after 10, 15, 20, 30, 45, 60, 90, 120, 180, 210, 240, and 270 min after injection. An enzymatic method was used to measure the serum concentration of sinistrin as described previously [24]. Cr was measured using a rate-blanked and compensated modified Jaffé method on a Cobas analyzer (Roche Diagnostics, Mannheim, Germany). The Cr assay was standardized by isotope-dilution using mass spectrometry (ID-MS). Liver tests were measured enzymatically and C-reactive protein (CRP) by immunoturbidometry (Roche Diagnostics, Mannheim, Germany). CysC was determined by particle-enhanced immunonephelometry using N Protein Standard UY from Siemens Healthcare Diagnostics, Marburg, Germany.

##### Statistical Analysis

Measured GFR (mGFR) was compared to eGFR determined by different Cr- and/or CysC-based equations (CG, MDRD4, Hoek, Larsson, CKD-EPI equations using Cr, CysC and both) (Additional file 1). For calculating the CG equation, the measured body weight of the patients was used. Agreement between mGFR and eGFR was assessed using the mean bias (average difference between mGFR and eGFR), standard error of the mean bias, the Pearson correlation coefficient and the concordance correlation coefficient as described previously [25–27]. Accuracy 10 % and accuracy 30 % of each equation were calculated [28]. Bland-Altman plots were prepared showing correlation and mean bias between mGFR and eGFR [29]. Patients’ characteristics were compared by Student’s *T* test for continuous variables and Fisher exact test for categorical ones. Accuracies (P10 and P30) and correlation coefficients were compared using McNemar’s test. Statistical analyses were performed using the commercial software SPSS (IBM SPSS Statistics 21) and STATA (Stata Statistical Software: Release 13. StataCorp, 2009, College Station, TX, USA).

## Results

### Patient characteristics and renal function of study cohort

Fifty cirrhotic patients and 24 age-matched healthy living kidney donors were studied. Of cirrhotic patients (78 % males, 22 % females), 18 (36 %) were classified as Child Turcotte Pugh (CTP) A, 18 as CTP B and 14 (28 %) as CTP C. Alcohol was the main cause of cirrhosis (72 %), followed by hepatitis C (8 %) and primary sclerosing cholangitis (8 %). The mean MELD score was 13 ± 5 (range 7–33). Amongst controls, more than half of the patients were female (75 %). Liver function in this group was normal. Patient characteristics are summarized in Table 1. Statistically significant differences between all cirrhotics and controls were found for total bilirubin, albumin, prothrombin time, CRP, and CysC (Table 1). The mean measured (m)GFR amongst all cirrhotic patients was 89.6 ± 27.5 mL/min/1.73 m^2^ and decreased with increasing cirrhosis severity (97.2 ± 24.1 mL/min/1.73 m^2^ - CTP A, 89.1 ± 25.2 mL/min/1.73 m^2^ - CTP B and 80.4 ± 32.8 mL/min/1.73 m^2^ - CTP C) (Table 2). Eight patients with cirrhosis had an mGFR < 60 mL/min/1.73 m^2^ (Additional file 2). Renal function in controls and CTP A patients was normal while it was mildly impaired (mGFR < 90 ml/min/1.73 m^2^) in cirrhotic patients at stage CTP B and C (Table 2).

**Table 1:** Characteristics of the study population

Characteristics	All Cirrhotics	CTP A	CTP B	CTP C	Controls
	[N=50]	[N=18 (36%)]	[N=18 (36%)]	[N=14 (28%)]	[N=24]
Males/Females	39/11	13/5 ^¥^	15/3 ^&^	11/9	6/18^¥^^&^
Age (yrs)	50 ± 9 (24–68)	47 ± 10 (24–67)	51 ± 8 (35–68)	54 ± 8 (36–65)	51 ± 11 (24–66)
BMI (kg/m2)	26 ± 5 (17–40)	25 ± 4 (20–32)	27 ± 6 (17–41)	27 ± 3 (21–34)	25 ± 5 (18–37)
MELD Score	13 ± 5 (7–33)	9 ± 2 (7–13) ^#^	12 ± 2 (8–18) §	19 ± 6 (12–33) ^# §^	n.a.
- MELD < 15	38 (76%)	18 (100%)	17 (94%)	3 (21%)	
- MELD > 15	12 (24%)	0 (0%)	1 (6%)	11 (79%)	
Etiology					n.a.
- Alcohol	36 (72%)	10 (56%)	13 (72%)	13 (93%)	
- Hepatitis B	1 (2%)	1 (6%)	-	| -	
- Hemochromatosis	1 (2%)	1 (6%)	-	I -	
- PSC	4 (8%)	3 (17%)	1 (6%)	| -	
- Unknown	1 (2%)	-	1 (6%)	| -	
- AIH	1 (2%)	1 (6%)	-	1 -	
- Hepatitis C	4 (8%)	1 (6%)	2 (11%)	1 (7%)	
- Wilson's disease	2 (4%)	1 (6%)	1 (6%)	| -	
Ascites					n.a.
- None	24 (48%)	17 (94%) ^% #^	6 (33%) ^%^	1 (7%) ^#^	
- Mild	11 (22%)	1 (6%) ^%^	8 (44%) ^%^	2 (14%)	
- Moderate	15 (30%)	-	4 (22%) ^§^	11 (79%) ^§^	
Hepatic Encephalopathy					n.a.
- None	43 (86%)	18 (100%)	17 (94%) ^§^	8 (57%) ^§^	
- Stage I-II	6 (12%)	-	1 (6%)	5 (36%)	
- Stage III-IV	1 (2%)	-	-	1 (7%)	
Portal Hypertension	46/50 (92%)	14/18 (78%)	18/18 (100%)	14/14 (100%)	n.a.
Total Bilirubin (mg/dL)	3.4 ± 6.1 (0.4-36.5)*	1.1 ± 0.6 (0.5-2.6) ^#^	1.9 ± 1.3 (0.4-6.4) ^§^	8.4 ± 10.1 (2.2-36.5) ^# §$^	0.5 ± 0.2 (0.2-1.5) * ^$^
Albumin (g/dL)	3.6 ± 0.7 (2.3-5.2)*	4.4 ± 0.3 (4.0-5.2)^% #^	3.4 ± 0.5 (2.8-4.5) ^% &^	3.1 ± 0.5 (2.3-3.8)^#$^	4.5 ± 0.4 (3.7-5.2) * ^&$^
Prothrombin Time (%)	62 ±18 (32–104)*	77 ± 14 (52–104)^% #^^¥^	61 ± 13 (42–88) ^% §&^	45 ± 12(32–73)^# §$^	99 ± 10 (72–114) * ^¥^^& $^
CRP (mg/dL)	9 ± 12 (3–70)*	3 ± 3 (3–11) ^#^	9 ± 8 (6–27)	19 ±19(3–70)^# $^	2 ± 2 (3–9) * ^$^
Cr (mg/dL)	0.8 ± 0.5-1.6)	0.8 ± 0.2 (0.6-1.4)	0.7 ± 0;2 (0;5–1;1)	0.9 ± 0.3 (0.5-1.6)	0.8: ± 0. 1(0.6–1.0)
CysC (mg/dL)	1.1 ±0.5 (0.6-3.9)*	1.0 ± 0.4 (0.6-2.4)	0.9 ± 0.3 (0.7-1.7)	1.5 ± 0.8 (0.5-3.9) ^$^	0.7 ± 0.1 (0.5-1.0)* ^$^

Values are expressed as means ± standard deviations or n (%); AIH, autoimmune hepatitis; BMI, body mass index; Cr, Creatinine; CRP, C-reactive protein; CTP, Child-Turcotte-Pugh Score; CysC, Cystatin C; MELD, model for end-stage liver disease; n.a., not applicable; PSC, primary sclerosing cholangitis. ^*^ p <0.05; statistical significant difference between cirrhotics (all) and controls, ^**^ p <0.05; statistical significant difference between CTP A and CTP B, ^***^ p <0.05; statistical significant difference between CTP A and CTP C, ^****^ p <0.05; statistical significant difference between CTP B and CTP C, ^*****^ p <0.05; statistical significant difference between CTP A and control, ^******^ p <0.05; statistical significant difference between CTP B and control, ^*******^ p <0.05; statistical significant difference between CTP C and control

**Table 2:** Performance of the different eGFR equations

Performance	CG	MDRD	CKD-EPI-Cr	Hoek	Larsson	CKD-EPI CysC	CKD-EPI-Cr-CysC
*All Cirrhotics* [*N* = 50] mGFR 89.6 ± 27.5							
eGFR	130.4 ± 41.0	101.1 ± 26.5	99.0 ± 18.5	78.0 ± 25.8	81.0 ± 31.3	80.9 ± 29.0	89.0 ± 24.8
Bias	−40.8 ± 29.2	−11.5 ± 22.0	−9.4 ± 20.7	11.1 ± 15.8	8.1 ± 17.7	8.2 ± 17.7	0.1 ± 16.3
Accuracy 10 %	4 %*	36 %	38 %	39 %	41 %	41 %	49 %
Accuracy 30 %	36 %*	74 %	78 %	84 %	82 %	84 %	84 %
CC (Pearson) (95 % CI)	0.703 (0.52–0.82)	0.688 (0.50–0.81)	0.658 (0.46–0.79)	0.862 (0.77–0.92)	0.826 (0.71–0.89)	0.805 (0.68–0.88)	0.812 (0.69–0.89)
CCC (95 % CI)	0.383^*^ (0.25–0.52)	0.611^*^ (0.45–0.77)	0.563^*^ (0.40–0.72)	0.757 (0.64–0.87)	0.789 (0.68–0.89)	0.771 (0.66–0.88)	0.807 (0.70–0.90)
CTP A [*N* = 18] mGFR 97.2 ± 24.1							
eGFR	126.6 ± 37.9	100.0 ± 26.1	99.7 ± 19.2	83.8 ± 25.2	88.0 ± 31.0	88.1 ± 27.6	93.8 ± 24.6
Bias	−29.4 ± 21.6	−2.8 ± 16.1	−2.5 ± 15.4	13.3 ± 12.3	9.2 ± 15.3	9.1 ± 12.6	3.4 ± 11.1
Accuracy 10 %	6 %	61 %	50 %	39 %	44 %	39 %	67 %
Accuracy 30 %	56 %	89 %	94 %	89 %	89 %	94 %	94 %
CC (Pearson) (95 % CI)	0.851 (0.64–0.94)	0.798 (0.53–0.80)	0.771 (0.48–0.91)	0.877 (0.69–0.95)	0.875 (0.69–0.95)	0.889 (0.72–0.96)	0.896 (0.74–0.96)
CCC (95 % CI)	0.529* (0.32–0.74)	0.790 (0.61–0.97)	0.746 (0.54–0.95)	0.785 (0.58–0.93)	0.801 (0.65–0.95)	0.827 (0.69–0.97)	0.887 (0.78–0.99)
CTP B [*N* = 18] mGFR 89.1 ± 25.2							
eGFR	142.7 ± 45.1	106.3 ± 22.7	102.9 ± 13.4	83.2 ± 20.4	86.7 ± 24.7	88.3 ± 25.3	95.4 ± 20.2
Bias	−53.7 ± 31.9	−17.2 ± 23.3	−13.8 ± 20.6	5.9 ± 20.1	2.3 ± 21.7	0.8 ± 21.7	−6.3 ± 19.1
Accuracy 10 %	0 %	22 %	33 %	44 %	44 %	44 %	39 %
Accuracy 30 %	28 %	67 %	78 %	83 %	78 %	78 %	78 %
CC (Pearson) (95 % CI)	0.725 (0.39–0.89)	0.534 (0.09–0.80)	0.581 (0.16–0.82)	0.628 (0.23–0.85)	0.623 (0.22–0.84)	0.632 (0.23–0.85)	0.667 (0.29–0.87)
CCC (95 % CI)	0.288 (0.10–0.48)	0.417 (0.10–0.74)	0.387 (0.11–0.66)	0.594 (0.30–0.89)	0.620 (0.32–0.92)	0.631 (0.34–0.93)	0.625 (0.35–0.90)
CTP C [*N* = 14] mGFR 80.4 ± 32.8							
eGFR	119.2 ± 37.9	95.8 ± 31.9	93.1 ± 22.7	62.9 ± 32.8	63.2 ± 35.2	60.5 ± 27.5	73.3 ± 25.6
Bias	−38.8 ± 29.4	−15.4 ± 24.8	−12.7 ± 25.4	15.1 ± 12.2	14.7 ± 12.5	17.4 ± 13.5	4.6 ± 16.3
Accuracy 10 %	7 %	21 %	29 %	31 %	31 %	38 %	38 %
Accuracy 30 %	21 %	64 %	57 %	77 %	77 %	77 %	77 %
CC (Pearson) (95 % CI)	0.663 (0.20–0.88)	0.706 (0.28–0.90)	0.635 (0.16–0.87)	0.930 (0.79–0.98)	0.934 (0.80–0.98)	0.915 (0.75–0.97)	0.872 (0.64–0.96)
CCC (95 % CI)	0.399 (0.10–0.69)	0.629 (0.32–0.94)	0.535 (0.20–0.87)	0.816 (0.66–0.98)	0.846 (0.70–0.99)	0.764 (0.57–0.96)	0.835 (0.67–0.99)
Controls [*N* = 24] mGF: 97.5 ± 15.1							
eGFR	98.7 ± 21.9	85.4 ± 12.9	93.5 ± 12.2	108.4 ± 17.9	118.9 ± 24.1	107.7 ± 13.6	102.1 ± 12.1
Bias	−1.2 ± 19.8	12.1 ± 12.3	4.1 ± 10.6	−10.9 ± 19.5	−21.4 ± 24.2	−10.2 ± 12.1	−4.5 ± 9.5
Accuracy 10 %	21 %*	16 %*	54.%	17 %*	37 %	54 %	71 %
Accuracy 30 %	92 %	100 %	100 %	83 %	62 %*	87 %	91 %
CC (Pearson) (95 % CI)	0.481 (0.09–0.74)	0.622 (0.29–0.82)	0.718 (0.44–0.87)	0.314^*^ (−0.10–0.64)	0.303^*^ (−0.11–0.62)	0.649 (0.33–0.83)	0.778 (0.54–0.89)
CCC (95 % CI)	0.449 (0.14–0.80)	0.443 (0.19–0.68)	0.671 (0.46–0.88)	0.252^*^ (−0.06–0.57)	0.171^*^ (−0.06–0.40)	0.510 (0.26–0.76)	0.718 (0.53–0.90)

mGFR and eGFR are expressed as means ± standard deviation in mL/min/1.73 m^2^; CC, correlation coefficient; CCC, concordance correlation coefficient. * *p* < 0.05; statistical significant differences between CDK-EPI-Cr-CysC and other eGFR equations

### Performance of Cr-based GFR equations in cirrhosis

**Fig. 1:**
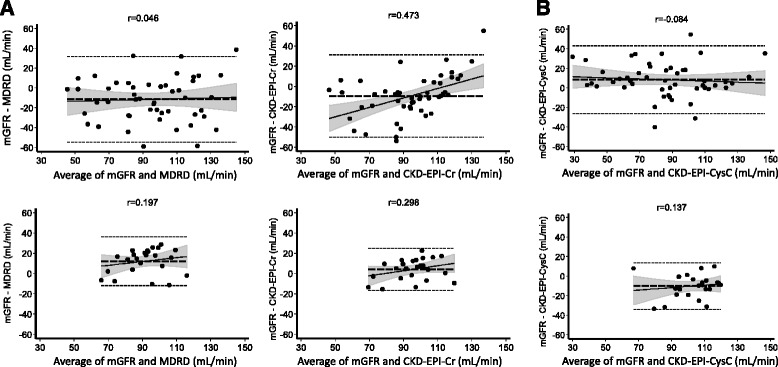
**a-b**. Bland-Altman-plots of eGFR determined by Creatinine (Cr)- and Cystatin C (CysC)-based equations. **a** Compared to mGFR, the commonly used Cr-based equations MDRD and CKD-EPI-Cr show less agreement with the gold standard in cirrhotic patients (**a**, upper panel) as compared to healthy controls (**a**, lower panel). **b** Compared to the Cr-based equations MDRD and CKD-EPI-Cr, the CKD-EPI-CysC equation shows a better performance in cirrhosis and an almost similar agreement with sinistrin clearance in cirrhotic patients (**b**, upper panel) and healthy controls (**b**, lower panel). **a**-**b** Horizontal long-dashed lines depict the bias (average difference between mGFR and eGFR), short-dashed lines show the limits of agreement between mGFR and eGFR according to the Bland-Altman method, and filled circles denote the measurement points. The oblique straight lines show the regression line between difference and average, and the shaded areas show the hyperbolic 95 % confidence limits of this regression line. R-values represent the Pearson’s linear correlation coefficients between difference and average. *MDRD*, Modification of Diet in Renal Disease; *CKD-EPI*, Chronic kidney disease epidemiology collaboration; *Cr*, Creatinine; *CysC*, Cystatin C

Cr-based equations overestimated mGFR in cirrhotic patients. Amongst all Cr-based equations, the CG equation showed the highest bias (−40.8 ± 29.2 mL/min/1.73 m^2^), followed by MDRD (−11.5 ± 22.0 mL/min/1.73 m^2^) and CKD-EPI-Cr (−9.4 ± 20.7 mL/min/1.73 m^2^). In line with the high bias, Cr-based equations in cirrhosis showed low 10 % (P10) and 30 % (P30) accuracies, defined as percentage of estimates within 10 % and 30 % of mGFR (4 % and 36 % for CG, 36 % and 74 % for MDRD and 38 % and 78 % for CKD-EPI-Cr). In healthy controls, accuracies of Cr-based equations were higher (Table 2), with exception of MDRD. Bland-Altman-plots of the two most commonly used Cr-based equations (MDRD, CKD-EPI-Cr) compared to mGFR are shown in Fig. 1A.

### Performance of CysC-based GFR equations in cirrhosis

In contrast to Cr-based equations, CysC-based equations underestimated mGFR. Amongst all CysC-based equations, the Hoek formula had the highest bias (11.1 ± 15.8 mL/min/1.73 m^2^), followed by CKD-EPI-CysC (8.2 ± 17.7 mL/min/1.73 m^2^) and the Larsson formula (8.1 ± 17.7 mL/min/1.73 m^2^). However, compared to Cr-based equations, CysC-based ones showed a better performance. With respect to P10 and P30, all three formulas were comparable with 39 % and 84 % for Hoek, 41 % and 82 % for Larsson and 41 % and 84 % for CKD-EPI-CysC. Underestimation of mGFR was especially found in CTP C (Table 2).

In controls, the performance of the Hoek, Larsson and CKD-EPI-CysC equation was not superior to Cr-based equations. Bias, P10 and P30 of the Hoek and Larson formula even showed worse performance compared to Cr-based equations and overestimated mGFR. Bland-Altman-plots comparing the most commonly used CysC-based equation (CKD-EPI-CysC) to mGFR are shown in Fig. 1B.

### Performance of Cr-based equations decreased with increasing Child-Turcotte-Pugh Score while performance of CysC-based equations remained rather constant

By increasing CTP score, performance of Cr-based equations decreased, showing high biases and low accuracies in patients with decompensated cirrhosis at stage CTP B and C (e.g. CKD-EPI-Cr: bias −13.8 ± 20.6 mL/min/1.73 m^2^, P10 33 % and P30 78 % for CTP B and bias −12.7 ± 25.4 mL/min/1.73 m^2^, P10 29 % and P30 57 % for CTP C) when compared to compensated cirrhotics at stage CTP A (CKD-EPI-Cr: bias −2.5 ± 15.4 mL/min/1.73 m^2^, P10 50 % and P30 94 % for CTP A) (Table 2). Although still overestimating mGFR, the CKD-EPI-Cr formula showed the best performance amongst all Cr-based equations at advanced stages of cirrhosis (CTP B and C). Compared to Cr-based equations, the performance of CysC-based ones was less influenced by increasing CTP score (e.g. CKD-EPI-CysC: bias 9.1 ± 12.6 mL/min/1.73 m^2^ in CTP A; bias 0.8 ± 21.7 mL/min/1.73 m^2^ in CTP B; bias −17.4 ± 13.5 mL/min/1.73 m^2^ in CTP C) (Table 2).

### Superiority of the CKD-EPI equation combining Cr and CysC in patients with cirrhosis

**Fig. 2:**
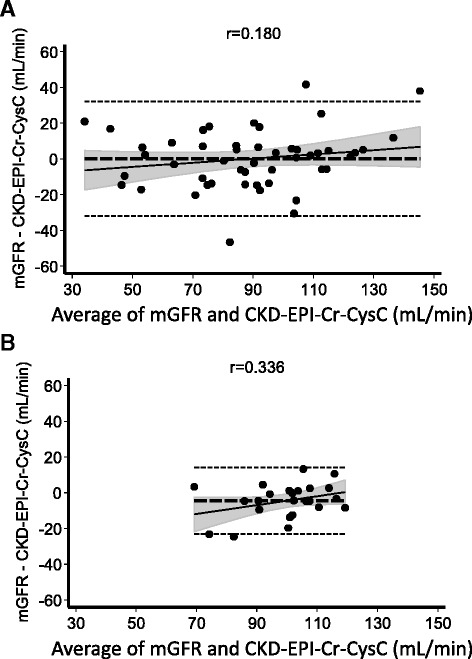
Bland-Altman-plots of eGFR determined by the combined CKD-EPI-Cr-CysC equation. Bland-Altman-plots of the CKD-EPI-Cr-CysC equation compared to mGFR determined by sinistrin clearance show an excellent agreement between eGFR and mGFR for the combined equation. The agreement between mGFR and eGFR using the combined formula in cirrhotic patients (**a**) is as good as that in healthy controls (**b**). **a**-**b** Horizontal long-dashed lines depict the bias (average difference between mGFR and eGFR), short-dashed lines show the limits of agreement between mGFR and eGFR according to the Bland-Altman method, and filled circles denote the measurement points. The oblique straight lines show the regression line between difference and average, and the shaded areas show the hyperbolic 95 % confidence limits of this regression line. R-values represent the Pearson’s linear correlation coefficients between difference and average. *CKD-EPI*, Chronic kidney disease epidemiology collaboration; *Cr*, Creatinine; *CysC*, Cystatin C

In patients with cirrhosis, the CKD-EPI formula combining Cr and CysC showed the best performance amongst all equations. Mean bias was low (0.1 ± 16.3 mL/min/1.73 m^2^) and 49 % of estimates were within 10 %, and 84 % were within 30 % of mGFR. Also at advanced stages of cirrhosis, low biases and high accuracies (bias −6.3 ± 19.1 mL/min/1.73 m^2^, P10 39 % and P30 78 % in CTP B and bias 4.6 ± 16.3 mL/min/1.73 m^2^, P10 38 % and P30 77 % in CTP C) were observed (Table 2). Due to the relatively low number of study patients, statistical significant differences for P10 and P30 between the CKD-EPI-Cr-CysC equation and the other equations were only found for CG (p < 0.0001 for P10 and P30 determined by McNemar’s test, Table 2). Also for correlation coefficients, statistical significant differences between CKD-EPI-Cr-CysC and all other eGFR equations were only found for Cr-based equations in all cirrhotics, CG in CTP A and CysC-based equations in healthy controls. Bland-Altman-plots comparing mGFR to eGFR determined by the CKD-EPI-Cr-CysC equation are shown in Fig. 2. Of 50 cirrhotics, 8 had an mGFR < 60 ml/min/1.73 m^2^ (Additional file 2). The combined CKD-EPI-Cr-CysC equation correctly identified 7 out of those 8 patients as patients with impaired renal function (eGFR < 60 ml/min/1.73 m^2^). In contrast, less than half of those patients were correctly identified using Cr-based equations and even a greater proportion of patients were found to have a GFR < 60 ml/min/1.73 m^2^ by using CysC-based equations (Additional file 3).

## Discussion

There is an urgent need for early and precise detection of impaired GFR in cirrhotic patients, especially in those suffering from acute on chronic liver failure (ACLF) and being evaluated for liver transplantation. We, therefore, prospectively evaluated GFR in 50 patients with cirrhosis and 24 healthy controls by measuring the renal function using sinistrin clearance (mGFR) and comparing its value with estimated (e)GFR using creatinine (Cr)- and Cystatin (Cys)C-based equations. We found that Cr- and CysC-based equations were inaccurate for the assessment of renal function in cirrhotic patients. Patients with known renal disease, diabetes or nephrotoxic drugs were excluded in order to exclusively study patients with impaired renal function most likely due to their liver disease. Only by the use of the combined CKD-EPI equation (CKD-EPI-Cr-CysC) we could obtain results comparable to those measured by sinistrin clearance. This represents the first prospective study with direct comparison of several equations for estimating GFR with the determination of GFR by measurement of sinistrin clearance using the bolus method.

Due to various limitations (e.g. malnutrition, muscle atrophy) the commonly used Cr-based equations for estimating renal function in cirrhosis are unreliable. Although having been considered a more sensitive indicator of renal function in cirrhosis [25, 30–34], CysC is influenced by factors independent of GFR which are frequently present in patients with cirrhosis such as elevated CRP or low serum albumin levels [15]. Since direct measurement of GFR by inulin clearance is technically demanding, time-consuming and costly, easier techniques using synthetic inulin-like polyfructosans (e.g. sinistrin) have been developed [17, 18]. In contrast to previous studies we determined mGFR by bolus intravenous injection of sinistrin, a simpler method without urine collection, which is considered to be more precise compared to the constant infusion standard method in healthy subjects and in patients with ascites.

Apart from severity of liver disease, which was less pronounced in our study cohort, patients’ characteristics were well comparable to previous studies in terms of age, gender and etiology of cirrhosis. Accordingly, we showed that Cr-based equations tend to overestimate mGFR and therefore could not serve as reliable parameters for assessing renal function in cirrhosis (Table 2). The accordance between eGFR and mGFR was much better in healthy controls, although the number of patients in this group was low. CysC-based equations rather underestimated mGFR in cirrhosis, however, the performance of these equations seemed to be less influenced by CTP score and was better compared to Cr-based ones, which has as well been confirmed by other studies [25, 32]. Although showing a better performance in accurately assessing mGFR than Cr-based equations, underestimation of GFR in end-stage cirrhotic patients by the use of CysC-based equations bears the potential risk of unnecessary simultaneous liver and kidney transplantation. Amongst all formulas, the CKD-EPI formula combining both, Cr and CysC, showed by far the best performance in cirrhosis (Table 2). Its diagnostic performance in cirrhotic patients was even as good as in healthy subjects and was independent of CTP score (Table 2). We confirmed the results of a recently published study that compared the performance of the CKD-EPI-Cr-CysC equation to mGFR determined by non-radiolabeled iothalamate plasma clearance in cirrhosis and found – similar to our study - that this equation was superior to other frequently used Cr- and CysC-based equations, although its performance was still worse than reported by others for non-cirrhotics [34]. However, measurement of GFR by renal clearance of iothalamate has been suggested to be not as accurate as compared to the gold standard inulin or sinistrin [35]. The performance of the CKD-EPI-Cr-CysC equation was superior in our study with only 16 % of the estimated GFR values differing from the mGFR by more than 30 % in cirrhotic patients (24 % in the study by Mindikoglu et al.) [34]. Our findings of decreasing accuracy of Cr-based formulas with the increase of CTP score are in line with results from a study by de Souza et al., who evaluated a dataset of 202 consecutive liver transplantation candidates. They described a better performance of CysC-based equations with CKD-EPI-CysC being considered as the most accurate equation whatever the magnitude of ascites and even in the presence of significant renal dysfunction. However, diabetics were also included and GFR was measured by the continuous infusion method, which has been reported to be inferior to the bolus method we used [17, 19, 36].

The main limitation of this study is the number of study patients that is relatively small. Due to that small number which was due to careful selection of included patients, only a few patients appeared to have renal dysfunction and only slight differences in the performance of CKD-EPI-Cr-CysC as compared to the other CKD-EPI equations were observed in patients with more advanced cirrhosis.

## Conclusions

We found that Cr-based equations were inaccurate to assess renal function in cirrhosis, in agreement with other studies [37, 38]. In general, CysC-based equations showed a better performance than Cr-based ones. Amongst all, the CKD-EPI equation combining Cr and CysC was superior to other equations in accurately assessing GFR in cirrhosis. Our results show the utility of cross validation of different tests to determine renal function in patients with advanced liver disease.

## Additional files

Additional file 1 GFR equations used in the study. Cr, serum creatinine (mg/dL); CysC, serum Cystatin C (mg/dL). (JPG 58 kb) Additional file 2 Characteristics of 8 cirrhotic patients with mGFR < 60 ml/min/1.73 m^2^. CTP, Child Turcotte Pugh Score; mGFR, measured glomerular filtration rate; PSC, primary sclerosing cholangitis. (JPG 27 kb) Additional file 3 Number of patients with GFR < 60 ml/min/1.73 m^2^ determined by IC and different Cr- and CysC-based eGFR equations. Of 50 cirrhotic patients, 8 had a GFR < 60 ml/min/1.73 m^2^. Only half of them or less were correctly identified by the use of creatinine-based equations (CG, MDRD, CKD-EPI-Cr). In contrast, even more patients were identified to have a GFR < 60 ml/min/1.73 m^2^ by using cystatin-C-based equations (Hoek, Larsson, CKD-EPI-CysC), again underlining the underestimation of GFR by the use of these equations. The combined CKD-EPI-Cr-CysC equation identified most of the patients with impaired renal function correctly compared to the gold standard. (PPTX 75 kb)

## Abbreviations

Cr: Creatinine
GFR: Glomerular filtration rate
CG: Cockcroft-Gault
MDRD: Modification of Diet in Renal Disease
CKD-EPI: Chronic Kidney Disease Epidemiology Collaboration
CysC: Cystatin C
mGFR: Measured glomerular filtration rate
eGFR: Estimated glomerular filtration rate
CTP: Child Turcotte Pugh
MELD: Model for end-stage liver disease
AIH: Autoimmune hepatitis
PSC: Primary sclerosing cholangitis
ALT: Alanine aminotransferase
AP: Alkaline phosphatase
BMI: Body mass index
CRP: C-reactive protein
