# Supplementary figures and images for: The chronic kidney disease epidemiology collaboration equation combining creatinine and cystatin C accurately assesses renal function in patients with cirrhosis
Source: BMC Nephrol. 2015 Dec 1;16:196. doi: 10.1186/s12882-015-0188-0 (PMC4665875; doi:10.1186/s12882-015-0188-0)

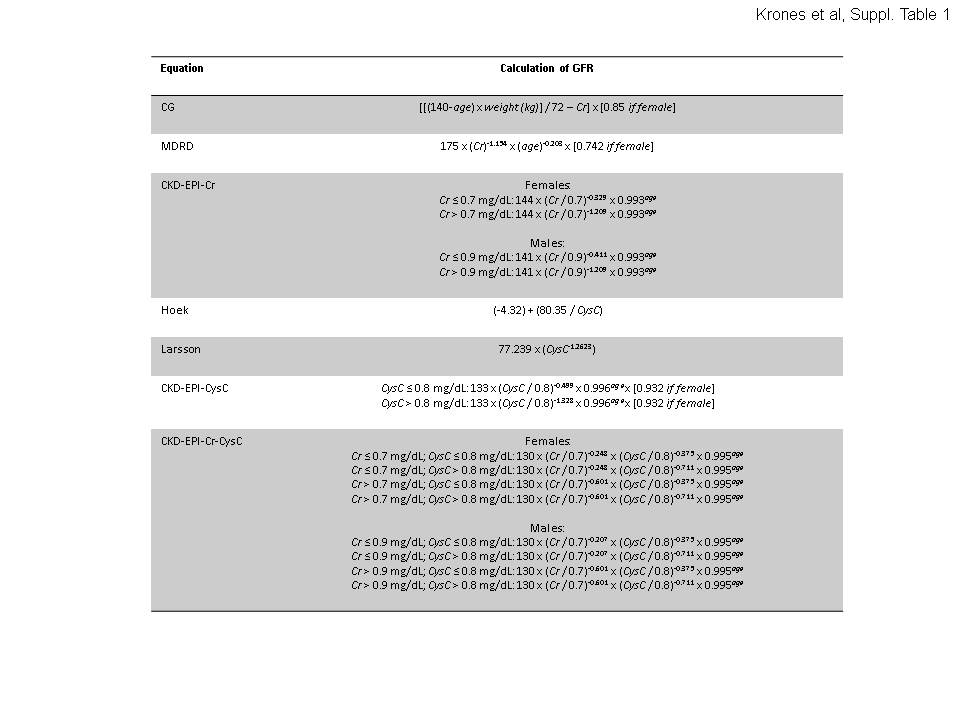

Supplement: Additional file 1 — GFR equations used in the study. Cr, serum creatinine (mg/dL); CysC, serum Cystatin C (mg/dL). (JPG 58 kb) [file 12882_2015_188_MOESM1_ESM.jpg]

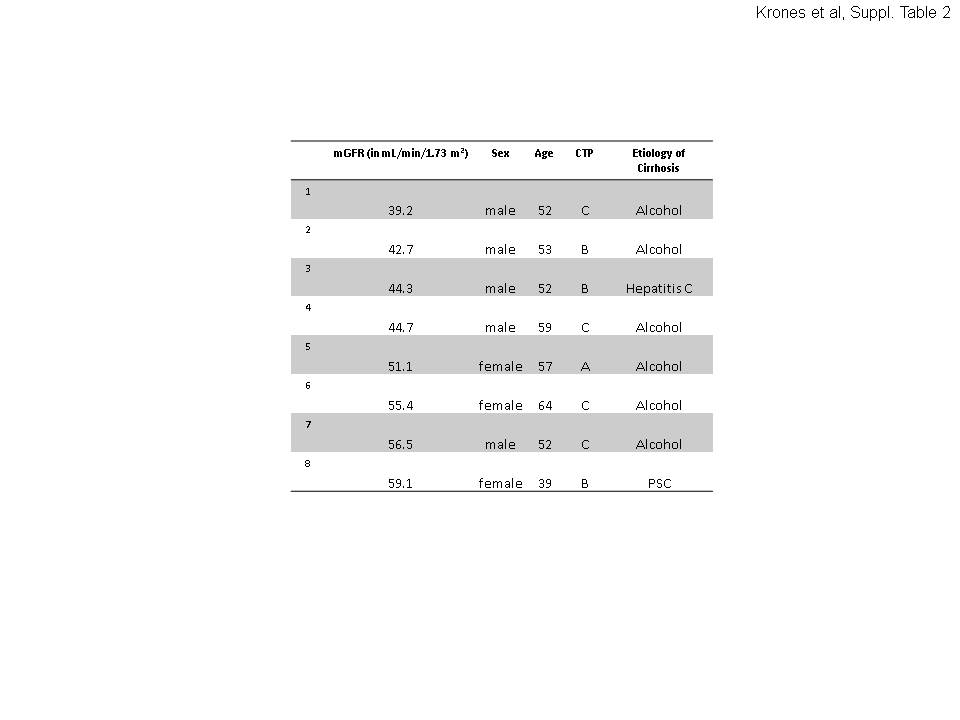

Supplement: Additional file 2 — Characteristics of 8 cirrhotic patients with mGFR < 60 ml/min/1.73 m2. CTP, Child Turcotte Pugh Score; mGFR, measured glomerular filtration rate; PSC, primary sclerosing cholangitis. (JPG 27 kb) [file 12882_2015_188_MOESM2_ESM.jpg]

## Slide 1
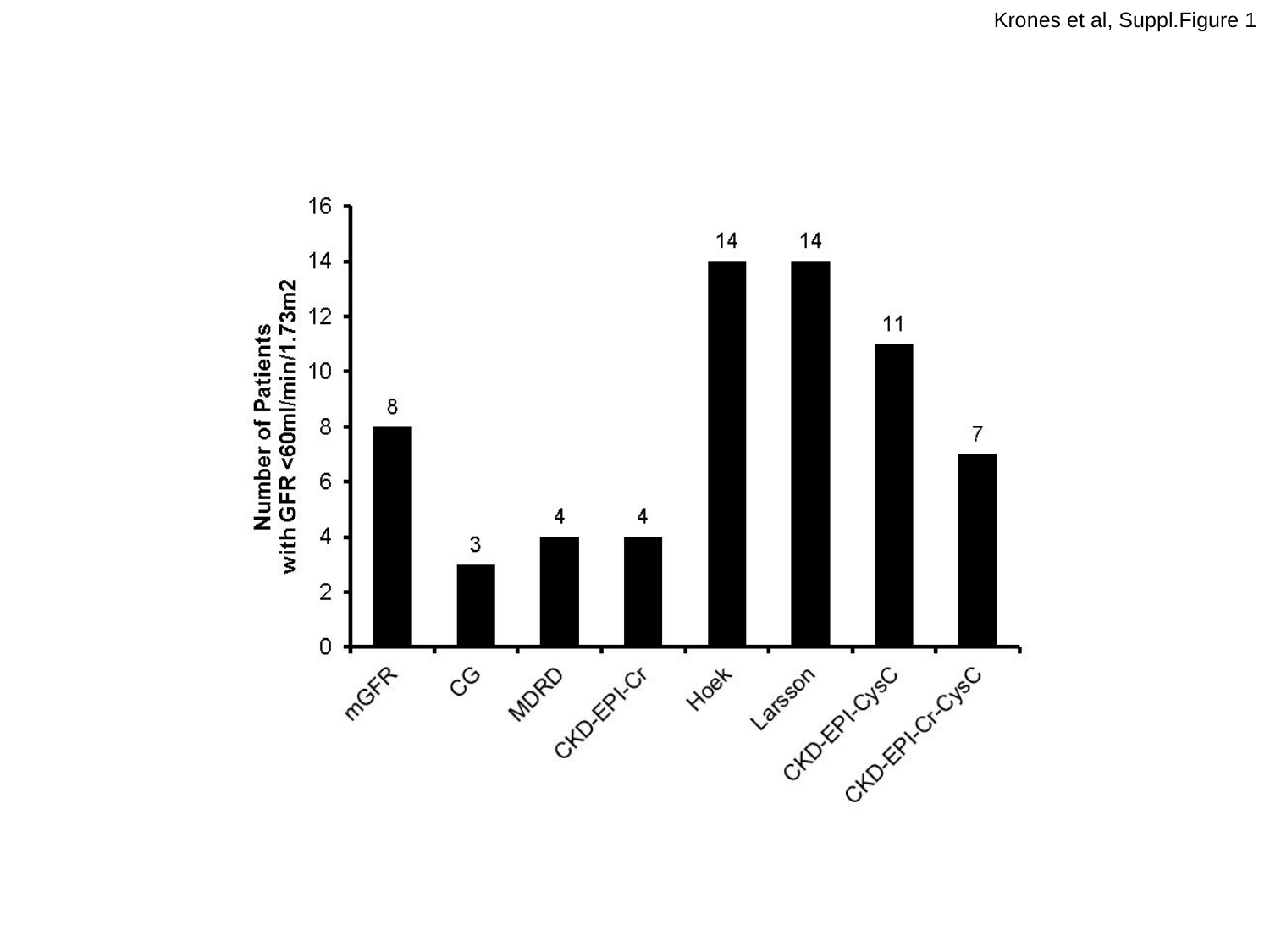

Krones et al, Suppl.Figure 1

Supplement: Additional file 3 — Number of patients with GFR < 60 ml/min/1.73 m2 determined by IC and different Cr- and CysC-based eGFR equations. Of 50 cirrhotic patients, 8 had a GFR < 60 ml/min/1.73 m2. Only half of them or less were correctly identified by the use of creatinine-based equations (CG, MDRD, CKD-EPI-Cr). In contrast, even more patients were identified to have a GFR < 60 ml/min/1.73 m2 by using cystatin-C-based equations (Hoek, Larsson, CKD-EPI-CysC), again underlining the underestimation of GFR by the use of these equations. The combined CKD-EPI-Cr-CysC equation identified most of the patients with impaired renal function correctly compared to the gold standard. (PPTX 75 kb) [file 12882_2015_188_MOESM3_ESM.pptx]
